# Lysosomal Regulation of mTORC1 by Amino Acids in Mammalian Cells

**DOI:** 10.3390/biom7030051

**Published:** 2017-07-07

**Authors:** Yao Yao, Edith Jones, Ken Inoki

**Affiliations:** 1 Life Sciences Institute, University of Michigan, 210 Washtenaw Avenue, Ann Arbor, MI 48109, USA; yaoyao@umich.edu (Y.Y.); jonesedi@umich.edu (E.J.); 2 Department of Molecular and Integrative Physiology, University of Michigan Medical School, 1137 East Catherine Street, Ann Arbor, MI 48109, USA; 3 Department of Internal Medicine, University of Michigan Medical School, 1500 East Medical enter Drive, Ann Arbor, MI 48109, USA

**Keywords:** mTOR, mTORC1, rapamycin, Rheb, Rag, TSC, lysosome, amino acid, growth factor

## Abstract

The mechanistic target of rapamycin complex 1 (mTORC1) is a master regulator of cell growth in eukaryotic cells. The active mTORC1 promotes cellular anabolic processes including protein, pyrimidine, and lipid biosynthesis, and inhibits catabolic processes such as autophagy. Consistent with its growth-promoting functions, hyper-activation of mTORC1 signaling is one of the important pathomechanisms underlying major human health problems including diabetes, neurodegenerative disorders, and cancer. The mTORC1 receives multiple upstream signals such as an abundance of amino acids and growth factors, thus it regulates a wide range of downstream events relevant to cell growth and proliferation control. The regulation of mTORC1 by amino acids is a fast-evolving field with its detailed mechanisms currently being revealed as the precise picture emerges. In this review, we summarize recent progress with respect to biochemical and biological findings in the regulation of mTORC1 signaling on the lysosomal membrane by amino acids.

## 1. Overview of Mechanistic Target of Rapamycin Complex 1

During evolution, cells in different species developed diverse strategies to sense extracellular cues and adapt to environmental changes. Among these extracellular cues, nutrient availability is the most fundamental element in determining cell survival and growth. In multicellular eukaryotic organisms, growth factor signaling has impinged on the nutrient signal to establish scrupulous regulation of cellular nutrient usage in a spatiotemporal manner. Thus, cells in different tissues systemically sense nutrients and use these signals to control their growth, proliferation, quiescence, or survival. Recent studies demonstrate that the cellular multiunit protein complex, mechanistic target of rapamycin (mTOR) complex 1, functions as a central regulator of cell growth in response to nutrients and growth factors.

mTOR is an evolutionarily conserved serine/threonine protein kinase, which belongs to the phosphoinositide-3-kinase (PI3K)-related family of protein kinases [1]. mTOR forms large protein complexes with other proteins, and the configurations of these mTOR complexes (mTORCs) are also evolutionarily conserved from yeast to mammals [2,3]. Two mTOR-containing multi-protein complexes have been identified, named mTOR complex 1 (mTORC1) and mTORC2 [4,5,6,7,8]. mTORC1 and mTORC2 have their specific accessory components: regulatory-associated protein of mTOR (Raptor) and proline-rich Akt substrate 40 (PRAS40) are specific to mTORC1 while the rapamycin-insensitive companion of mammalian target of rapamycin (Rictor), stress-activated protein kinase-interacting protein 1 (Sin 1), and the protein observed with Rictor-1 (Protor) are specific for mTORC2. Two other proteins, mammalian lethal with Sec13 protein 8 (mLST8) and Dishevelled, Egl-10, and Pleckstrin (DEP) domain containing mTOR interacting protein (Deptor), are common mTOR interacting proteins found in both mTORC1 and mTORC2 [9,10,11,12,13,14]. In addition, both mTORC1 and mTORC2 form an obligate dimer [15,16,17]. The major cellular role of mTORC1 involves its cell growth control, while mTORC2 regulates cytoskeleton organization and cell survival.

mTORC1 activity is sensitive to rapamycin, a macrolide originally developed as an antifungal agent [18]. Rapamycin strongly interacts with FK506-binding protein 12 (FKBP12), and this drug–protein complex binds to the FKBP12–rapamycin-binding (FRB) domain of mTOR kinase [19]. The FRB domain acts as a gatekeeper since its rapamycin binding site interacts with substrates to grant them access to the restricted active site of mTOR kinase. The rapamycin–FKBP12 complex therefore allosterically inhibits mTOR kinase by blocking substrate recruitment and further restricts the accessibility of substrates to the active site of mTOR kinase [20]. However, the sensitivity of rapamycin varies significantly among different mTORC1 substrates. Interestingly, the FKBP12–rapamycin complex not only restricts the accessibility of substrates to the active site of mTOR kinase but also weakens the mTOR–Raptor interaction and destabilizes the dimeric structure of mTORC1 [6,15,21]. It has been postulated that the dimeric conformation of mTORC1 is required for its phosphorylation of eukaryotic translation initiation factor 4E binding protein 1 (4EBP1) but not ribosomal S6 kinase 1 (S6K1) [15]. Thus, in addition to the size of substrates, the integrity of the mTORC1 dimer determines the sensitivity of rapamycin to inhibit mTORC1-dependent phosphorylation of its substrates.

Raptor is an essential component of mTORC1, forming an obligate dimer with an overall rhomboid shape and a central cavity. The dimeric interfaces are formed by interlocking interactions between mTOR and Raptor [15]. Raptor functions as a scaffold protein to recruit mTORC1 substrates such as S6K1 and 4EBP1. These substrates are recognized by mTORC1 through their TOR signaling (TOS) motif, which is a conserved five amino acid sequence and is crucial for their interaction with Raptor [22,23,24]. In addition to its role in substrate recognition, recent studies reveal the role of Raptor in determining mTORC1 subcellular localization. In response to amino acids, Raptor interacts with the lysosomal Ras-related guanosine 5’-triphosphate (GTP)-binding protein (Rag small GTPase protein complex tethering mTORC1 to the lysosomal membrane, where it encounters another small GTPase, Ras homolog enriched in brain (Rheb) that directly interacts with the mTOR kinase and stimulates the activity of mTORC1 [25,26]. 

Rheb resides at the cellular endomembrane system [26,27,28]. Previous studies suggested that Rheb localizes on different cellular compartments, including endoplasmic reticulum (ER), Golgi, mitochondria, peroxisome, and importantly, lysosome [29,30,31,32]. Rheb localizes to their membranes through its farnesylation on the “CAAX” motif [33]. The mutation of cysteine in the CAAX motif disrupts the membrane localization of Rheb and disables the ability of Rheb for mTORC1 activation, suggesting that appropriate membrane localization of Rheb and mTORC1 are required for mTORC1 activation. As a small GTPase protein, the GTP/guanosine 5'-diphosphate (GDP) loading status of Rheb is important for its activity. When GTP is loaded, Rheb functions as a potent stimulator for mTORC1 kinase activity [11,34]. However, the precise molecular mechanisms by which Rheb specifically stimulates mTORC1 have not been well understood. It has been demonstrated that the tuberous sclerosis complex (TSC) consisting of TSC1, TSC2, and the Tre2-Bub2-CDC16 (TBC) 1 domain family member 7 (TBC1D7) also localizes on the membrane of lysosomes and peroxisomes and inhibits Rheb activity by functioning as a GTPase activating protein (GAP) complex [31,35,36,37,38,39,40]. Among these subunits, TSC2 bears a GAP domain, which specifically converts Rheb from the GTP-bound active form to GDP-bound inactive form [35,41,42]. The activity of TSC2 is regulated by the growth factor-dependent PI3K–Akt pathway. Akt directly phosphorylates at least four serine and threonine residues of TSC2 and induces the dissociation of the TSC complex from lysosomal membranes [27,43]. Although the molecular mechanisms by which TSC2 phosphorylation by Akt dislocates the entire TSC complex away from the lysosome remain unclear, the absence of its GAP activity on the lysosomal membrane provides the permissive conditions for Rheb GTP-loading and its activation [27] (refer to the subsequent section for details). Interestingly, recent studies demonstrated that lysosomal localization of TSC is also diminished by amino acids [43,44] (see the details in the following section). Thus, the coordinated spatial regulations of both mTORC1 and the TSC complex establish the machinery for sensing multiple environmental cues to regulate cell growth control. 

## 2. The Lysosome is the Major Cellular Compartment for mTORC1 Activation

The lysosome is a major catabolic organelle and is responsible for the degradation of all kinds of biomolecules [45]. Over 60 digestive enzymes are found in the lysosomal lumen and are used for macromolecule hydrolysis (i.e., proteins, lipids, and polysaccharides) breaking them down into their constitutive monomers (i.e., amino acids). These digested monomers are exported to the cytosol from lysosomes via diffusion and/or through specific transporters as fuels for various metabolic and biosynthetic pathways in response to cellular demands. 

Although mTORC1 can be activated at the Golgi apparatus and the peroxisome [30,31,46], recent studies demonstrated that the lysosomal membrane is the major site for mTORC1 activation [25,26,47,48]. In response to amino acid availability, mTORC1 is recruited to the lysosomal membrane from unidentified cytosolic foci. The disruption of lysosomal mTORC1 localization largely diminishes its activation by amino acids. In contrast, artificially tethering mTORC1 to lysosomes renders constitutive mTORC1 activation, regardless of amino acid availability. This constitutive mTORC1 activation depends on Rheb, as its deletion abolishes mTORC1 activation even though mTORC1 is localized on the lysosomal membrane. Thus, the major role of amino acid input for mTORC1 activation is recruitment of mTORC1 to the place where Rheb, a direct activator of mTORC1, is localized. In support of this idea, tethering both mTORC1 and Rheb to other membrane compartments such as the plasma membrane sufficiently induces mTORC1 activation [25]. These observations clearly indicate that the lysosomal membrane functions as a key physiological platform to merge mTORC1 and Rheb for mTORC1 activation in response to amino acid availability.

## 3. Amino Acid-Sensing Signaling to the Lysosomal Membrane

### 3.1. The Rag GTPase and the Ragulator Complex Form a Super Complex with Vacuolar-ATPase, which Recruits mTORC1 to the Lysosomal Membrane in Response to Amino Acid Availability

Diverse upstream signals including growth factors, hypoxic stress, energy, and amino acids impinge on the TSC complex to regulate Rheb–mTORC1 activity. Among these signal inputs, amino acids also exert a crucial role in supporting mTORC1 activation independent of the regulation of the TSC complex. mTORC1 activity is still inhibited upon amino acid withdrawal while it is resistant to growth factor starvation in cells lacking functional TSC complex [49,50].

**Figure 1:**
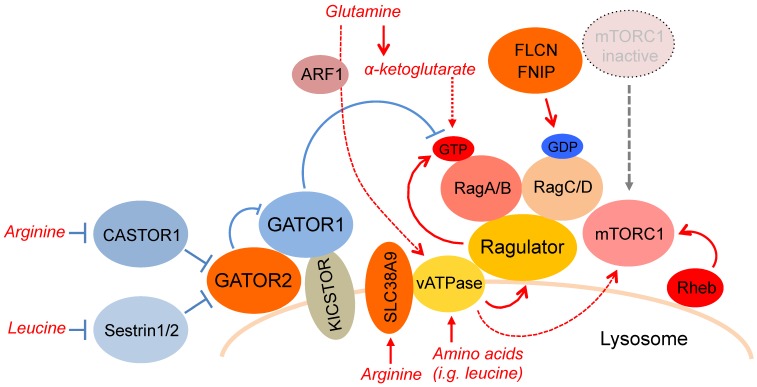
Amino acid-sensing mechanisms that recruit mechanistic target of mTORC1 to the lysosomal membrane. Cytosolic arginine and leucine activate GATOR2 by CASTOR1 and Sestrin1/2, respectively, leading to the inhibition of GATOR1, the GAP for the RagA/B small GTPases. Lysosomal luminal arginine activates vATPase through SLC38A9, leading to the activation of Ragulator, the guanine exchange factor (GEF) for RagA/B. Upon the activation of the Rag heterodimer, mTORC1 is recruited to the lysosomal membrane and is then activated by the small Rheb GTPase. CASTOR: cellular arginine sensor for mTORC1; Rheb: Ras homolog enriched in brain; GATOR: GTPase activating protein (GAP) activity toward Rags; KICKSTOR: Kaptin (KPTN), Integrin alpha phenylalanyl-glycyl-glycyl-alanyl-prolyl (FG-GAP) repeat containing 2 (ITFG2), chromosome 12 open reading frame 66 (C12orf66) and seizure threshold 2 homolog (SZT2)-containing regulator of mTORC1; Rag: Ras-related GTP binding; mTORC1: mechanistic target of rapamycin complex 1; GDP: guanosine 5’-diphosphate; GTP: guanosine 5’-triphosphate; ARF1: adenosine diphosphate-ribosylation factor 1; FLCN: folliculin; FNIP: folliculin interacting protein; vATPase: vacuolar H^+^-ATPase; SLC38A9: solute carrier family 38 member 9.

By using genetic and biochemical approaches, studies from different labs have identified that an evolutionarily conserved Ras-related small GTPase (Rag), plays a key role in enhancing mTORC1 activity in response to amino acids [26,51]. Mammalian cells contain four members of Rag proteins (RagA, B, C, and D), which are expressed on the lysosomal membrane [52]. RagA and B, like RagC and D, are highly similar to each other and functionally redundant. Rags form obligate heterodimers of either RagA or RagB with either RagC or RagD. Interestingly, in the active Rag heterodimer, RagA or RagB binds to GTP while RagC or RagDs binds to GDP. In addition, these nucleotide-loading states are tightly regulated by lysosomal luminal and cytoplasmic amino acids although the precise measurement of the in vivo nucleotide-loading status of each Rag small GTPase in the Rag heterodimer is challenging. Indeed, Oshiro et al. failed to detect any significant changes of GTP-charging toward RagA and RagC in response to amino acid stimulation [53]. Thus, further efforts to develop relevant methods (e.g., active/inactive-Rag antibodies) are necessary for monitoring in vivo Rag activity. Upon amino acid availability, the active Rag heterodimer interacts with mTORC1 through Raptor, an essential component of mTORC1, thereby recruiting mTORC1 to the lysosomal membrane [26]. Loss of functional Rag heterodimer largely disrupts lysosomal mTORC1 localization and significantly reduces acute induction of mTORC1 activity in response to amino acid stimulation. In contrast, the expression of a constitutive active Rag heterodimer (e.g., RagB–GTP/RagC–GDP) confers constitutive lysosomal mTORC1 localization with its activity being resistant to amino acid starvation (Figure 1).

Although the Rag heterodimer localizes on the lysosomal membrane, Rags do not possess membrane localization signals, unlike other small GTPases. Importantly, lysosomal Rag expression depends on Ragulator, a pentameric protein complex anchored on the lysosome [25,26,47]. Ragulator consists of p18 (late endosomal/lysoosmal adaptor and mitogen-activated protein kinase and mTOR activator 1 (LAMTOR1)), p14 (LAMTOR2), mitogen-activated protein kinase (MAPK) /extracellular signal-regulated kinase (ERK) kinase (MEK) binding partner 1 (MP1/LAMTOR3), C7ORF59 (LAMTOR4), and hepatitis B virus X-interacting protein (HBXIP/LAMTOR5) and interacts with the Rag heterodimer. One of the Ragulator subunits, LAMTOR1, is myristoylated and palmitoylated at its N-terminus and anchors the Ragulator complex and Rag heterodimer to the lysosomal membrane [25,47,54]. In support of this model, the Rag heterodimer is unable to localize on the lysosomal membrane in cells lacking LAMTOR1. As expected, amino acid-induced lysosomal mTORC1 localization and its activation are largely diminished in LAMTOR1 deficient cells as seen in RagA/B knockout cells. These observations indicated that the Ragulator complex is an essential component in Rag-dependent mTORC1 activation in response to amino acids (Figure 1).

Importantly, in addition to its scaffolding role for the Rag heterodimer on the lysosomal membrane, the Ragulator complex also functions as a GEF for RagA and RagB [47]. Upon amino acid stimulation, the GEF activity of Ragulator promotes GTP loading to RagA and RagB in a manner dependent on lysosomal vATPase activity. Moreover, four out of five components of Ragulator (LAMTOR2–5) contain a putative roadblock domain, which is often observed in GTPase regulatory proteins [55,56]. However, the precise molecular mechanism as to which each subunit plays a critical role for the Ragulator complex GEF activity remains elusive. As all of the components of Ragulator are indispensable for its function as a Rag A/B GEF, it is possible that a tertiary structure composed by these subunits is required for the GEF activity towards RagA/B. This specific GEF activity toward Rag A/B but not Rag C/D likely stems from the difference between the RagA/B and RagC switch I/II regions, which are known to be a critical recognition motif on a GTPase for its cognate GEF [57]. As a GEF preferentially interacts with GDP-bound or nucleotide-free small GTPases, Ragulator also binds to the nucleotide-free RagA/B mutant with higher affinity compared to the wild type RagA/B. Accordingly, the interaction between RagA/B and Ragulator is weakened by amino acid stimulation whereas it is strengthened by amino acid starvation. These observations raise the possibility that GTP-loading to RagA/B incurs a conformational change of the Rag–Ragulator complex, which has a higher affinity for mTORC1. Other possibilities include that the GTP–Rag heterodimer interacting with mTORC1 may dissociate from the Ragulator complex upon amino acid stimulation to ferry mTORC1 from the lysosomal membrane to the cytosol [58]. Further studies will be required to determine the molecular mechanisms underlying the dynamics of Ragulator–Rag–mTORC1 interactions in response to amino acid stimulation or starvation.

By using an in vitro system, Zoncu et al. proposed that the lysosome contains all the machinery required for mTORC1 recruitment in response to amino acids as amino acid stimulation in vitro induces the association of Raptor with immunopurified lysosomes [48]. Intriguingly, the study also demonstrated that the amino acids inside of the lysosomal lumen play a key role in initiating a signal for mTORC1 recruitment to the lysosomal membrane and subsequent mTORC1 activation. Zoncu et al. also demonstrated that vATPase directly interacts with Ragulator, and that the structural rearrangement of vATPase but not the lysosomal proton gradient is important for lysosomal mTORC1 localization and activation. Treatment of isolated lysosomes with Streptolysin O or Triton X-100, which induce lysosomal luminal leakage, largely inhibits amino acid-induced Raptor interaction with the Ragulator–Rag complex in vitro. Furthermore, cells overexpressing H+/amino acid transporter 1 (PAT1/SLC36A1), a lysosome-specific proton-coupled amino acid transporter that exports amino acids out of the lysosomal lumen, inhibit amino acid-induced mTORC1 activation, though physiological levels of PAT1 are required for intact lysosomal function and mTORC1 activation [59]. Based on these observations, a lysosome-centric inside-out model of amino acid-sensing by mTORC1 has been proposed. This model states that amino acids within the lysosomal lumen initiate the signal for mTORC1 recruitment to the lysosomal membrane through the vATPase–Ragulator–Rag complex [48] (Figure 1).

Although the above lysosome-centric inside-out model clearly demonstrated how RagA and RagB are GTP-loaded through the Ragulator complex by luminal amino acids in the lysosome, recent studies have also identified other non-lysosomal amino acid sensors such as Sestrins (leucine sensor) and CASTOR proteins (arginine sensor), which regulate RagA/B GTP hydrolysis through GATOR1 [60,61] (refer to Sestrin and CASTOR sections). It is important to note that while the lysosomal Rag heterodimer plays a pivotal role on amino acid-induced lysosomal mTORC1 localization and its acute activation, cells seem to possess alternative mechanisms to sense amino acid availability in a manner independent of the Ragulator–Rag system. For instance, glutamine is able to stimulate lysosomal mTORC1 localization and its activity in RagA/RagB double knockout cells in an ADP-ribosylation factor 1 (ARF1)-dependent manner [62]. Furthermore, amino acids also stimulate GTP-charging to Rab1A, which stimulates the interaction between Rheb and mTORC1 at the Golgi and mTORC1 activation [30]. The molecular mechanisms by which Arf1 and Rab1 sense amino acids remain elusive and need further investigations. 

### 3.2. GATOR1 is a GTPase Activating Protein for Rag A/B, while GATOR2 is a Negative Regulator of GATOR1

The Ragulator complex stimulates RagA/B through its GEF activity in response to lysosomal luminal amino acids. By contrast, recent studies identified GATOR, an octomeric protein complex, as a key regulator of RagA/B [63] (Figure 1). 

GATOR (GTPase-activating protein activity toward Rags) is composed of two sub-complexes, GATOR1 and GATOR2, and localizes on the lysosome. Three proteins, DEP domain containing 5 (DEPDC5), nitrogen permease regulator 2-like protein (Nprl2), and Nprl3 comprise GATOR1, which inhibits the activity of Rag A/B, while the pentameric GATOR2 complex consisting of protein subunits, meiosis regulator for oocyte development (Mios), tryptophan-aspartic acid (WD) repeat-containing protein 24 (WDR24), WDR59, SEH1-like nucleoporin (Seh1L), and Sec13, functions as a suppressor of GATOR1 through unknown molecular mechanisms [63]. Loss of functional GATOR1 confers mTORC1 resistance to amino acid starvation and allows for its constitutive lysosomal localization. In contrast, loss of functional GATOR2 renders mTORC1 insensitive to amino acid stimulation and unable to localize to the lysosomal membrane even under amino acid availability conditions. Importantly, GATOR1 complex possesses specific GAP activity toward Rag A/B.

Interestingly, mutations in genes encoding GATOR1 components such as DEPDC5 and Nprl2 have been found in several cancer cells such as astrocyte tumors with chromosome 22 rearrangements, lung cancers with homozygous deletion on chromosome region 3p21.3, and NPRL2/G1 homozygous deletion in renal, lung and cervical cell lines [64,65,66]. In these cancer cell lines, as expected, mTORC1 constitutively localizes to the lysosomal membrane and therefore maintains its activity even under amino acid starvation conditions. Re-introduction of intact GATOR1 into these cells renders mTORC1 sensitive to amino acid starvation. Notably, the proliferation of these cells with loss of function GATOR1 mutations is highly sensitive to the mTORC1 inhibitor rapamycin compared to other cancer cell lines including HeLa and PC3, which bear phosphatase and tensin homolog (PTEN) loss of function mutations. 

### 3.3. SLC38A9 is a Lysosomal Arginine Sensor for mTORC1 Activation

Among the twenty classical L-amino acids, arginine and leucine are two essential amino acids that potently stimulate the activity of mTORC1 in mammalian cells. However, the molecular mechanisms by which these specific amino acids stimulate mTORC1 activity have not been clearly understood. By searching amino acid transporters that localize on the lysosomal membrane or regulatory proteins that interact with the Ragulator–Rag complex, SLC38A9 was identified as a lysosomal amino acid transporter that interacts with the Ragulator–Rag complex [67,68]. SLC38A9 is a previously uncharacterized trans-membrane protein with sequence similarity to the SLC38 class of sodium coupled amino acid transporters [69]. SLC38A9 is predicted to have 11 trans-membrane domains with a cytosolic N-terminal region of 119 amino acids and a lysosomal luminal trans-membrane region [67,69]. SLC38A9 interacts with both the Ragulator complex and vATPase through its distinct regions on the lysosomal membrane [54,67]. Interestingly, the interaction between SLC38A9 and Ragulator is regulated by amino acid availability, as amino acid stimulation or starvation weakens or strengthens their interaction, respectively. Ablation of SLC38A9 suppresses the activation of mTORC1 by amino acids, whereas overexpression of wild-type SLC38A9 or the N-terminal 119 amino acids (Ragulator-binding domain) confers the mTORC1 activation resistant to amino acid starvation [67,68]. Epistatic analyses suggest that SLC38A9 functions upstream of the small Rag GTPases, as ectopic SLC38A9-induced mTORC1 activation is largely blocked by the expression of the dominant negative Rags [68]. These observations suggest that conformational rearrangements induced by amino acids between SLC38A9 and the Ragulator complex are necessary to stimulate the Ragulator–Rag system. Importantly, ablation of SLC38A9 specifically attenuated arginine-induced but not leucine-induced mTORC1 activation [67]. Taken together, the studies suggest that SLC38A9 functions as a lysosomal membrane-resident arginine sensor for mTORC1 activation. However, it remains unclear how SLC38A9 specifically conveys a signal from arginine to the vATPase–Ragulator–Rag complex. More recently, Castellano et al. demonstrated that SLC38A9 also interacts with cholesterol through its cholesterol recognition motifs within the trans-membrane domain 8 and Niemann-Pick C1 (NPC1), which regulates cholesterol export from the lysosome [70]. SLC38A9 is required for mTORC1 activation by cholesterol in a manner independent of its arginine-sensing function. In contrast, NPC1 binds to SLC38A9 and inhibits mTORC1 activity through its sterol transport function. Thus, SLC38A9 functions as a key sensor for both arginine and cholesterol availability to instigate mTORC1 activation through the vATPase–Ragulator–Rag system on the lysosomal membrane.

### 3.4. CASTOR Proteins are Cytosolic Arginine Sensors for mTORC1 Activation

Although SLC38A9 plays a key role in sensing arginine availability to stimulate mTORC1 through the Ragulator complex, a GEF for RagA/B on the lysosomal membrane, a recent study also identified the cytosolic arginine sensor, cellular arginine sensor for mTORC1 (CASTOR), which activates GATOR1, a GAP for RagA/B, by inhibiting GATOR2, the upstream suppressor of GATOR1 [60] (Figure 1). CASTOR was originally identified as a GATOR2 interacting protein [60]. In vertebrates, two CASTOR proteins (CASTOR1 and CASTOR2 also known as stromal antigen (STAG) 3 opposite strand protein like 3 (GATSL3) and GATSL2, respectively) are found as cytosolic proteins. Intriguingly, both CASTOR1 and CASTOR2 bear four tandem aspartate kinase, chorismate mutase and TyrA (ACT) domains [60,71], which are known to interact with diverse small molecules such as amino acids and nucleotides [72,73,74,75]. CASTOR1 and CASTOR2 form a homo- or heterodimer [60]. Interestingly, amino acid depletion significantly enhances the interaction between GATOR2 and the CASTOR complex containing CASTOR1, whereas amino acids induce the dissociation of these complexes. Critically, CASTOR1 but not CASTOR2 specifically binds to arginine with a dissociation constant (Kd) of approximately 35 µM, which is similar to the half maximal concentration of arginine that induces the dissociation of GATOR2 from CASTOR1 in vitro and activates mTORC1 in vivo. Overexpression of CASTOR1 largely inhibits amino acid-induced mTORC1 activation, whereas ablation of CASTOR1 in cells confers mTORC1 activity substantially insensitive to deprivation of arginine. Furthermore, the CASTOR1 mutant that is unable to interact with arginine constitutively binds to GATOR2, rendering mTORC1 insensitive to arginine stimulation [71]. These results suggest that arginine binding to CASTOR1 triggers its dissociation from GATOR2 and relieves CASTOR1’s inhibitory effect on GATOR2 (Figure 1). Notably, while dimerization of CASTOR1 is dispensable for arginine binding, CASTOR1 mutants are unable to form a dimer and only weakly interact with GATOR2 and lose their inhibitory effect on mTORC1 activity, indicating that dimerization of CASTOR1 is critical for its inhibitory effect on GATOR2. 

The structure of CASTOR1 revealed that CASTOR1 forms a dimer, consistent with observations in previous biochemical analyses [60]. Among the four ACT domains in each CASTOR1 monomer, ACT2 and ACT4 generate an arginine-binding pocket at the interface of these domains [71,76,77]. The bound arginine forms extensive hydrophilic and hydrophobic interactions with the surrounding residues composing the binding pocket. Importantly, the critical residues of CASTOR1 for its interaction with GATOR2 cluster along the surface of the ACT2–ACT4 interface, adjacent to the arginine-binding pocket. One of these important residues is buried deep in the ACT2–ACT4 interface in the arginine-bound conformation of CASTOR1, potentially explaining why GATOR2 is unable to interact with the arginine-bound form of CASTOR1. Taken together, SLC38A9 and CASTOR1 have unique subcellular localizations and receive arginine signals from different cellular compartments thus regulating mTORC1 through distinct molecular mechanisms. SLC38A9 localizes on the lysosome and likely senses lysosomal luminal arginine to activate the Ragulator–Rag pathway in a vATPase dependent manner. On the other hand, CASTOR1 senses cytosolic arginine to regulate the GATORs–Rag pathway. 

### 3.5. Sestrin2 is a Leucine Sensor for the mTORC1 Pathway, and Regulates the Activity of Rags through GATORs

Importantly, three recent independent studies have identified that Sestrins also interact with GATOR2 and inhibit mTORC1 activity [78,79,80] (Figure 1). The mammalian Sestrins comprise three related proteins, Sestrin1, 2 and 3. The expression of Sestrins is induced by several stress-responsive transcription factors such as p53, CCAAT/enhancer binding protein (C/EBP) bata, activating transcription factor 4 (ATF4), and forkhead box O proteins (FoxOs) [81,82,83]. Consistent with the roles of these transcription factors, Sestrins maintain cellular homeostasis in response to DNA damage, amino acid insufficiency, energy starvation, and oxidative stress [81,82,83]. Importantly, Sestrin1 and Sestrin2 strongly interact with GATOR2 under amino acid-deficient conditions while Sestrin3 constitutively interacts with GATOR2, irrespective of amino acid availability [61]. Furthermore, leucine is the only amino acid able to disrupt the interaction between Sestrin2 and GATOR2 within its physiological concentration both in vivo and in vitro. 20~40 µM leucine shows half-maximal effects on both the Sestrin2–GATOR2 interaction and mTORC1 activation in cultured cells. Moreover, Sestrin2 directly interacts with leucine but not arginine with a dissociation constant of ~20 µM. 

The structure of Sestrin2 revealed that Sestrin2 bears an evolutionarily unique leucine-binding pocket, which specifies a leucine with several hydrophobic residues and holds it with adjacent charged residues [84]. In addition, the bound-leucine is concealed by the hydrophilic threonine residues adjacent to the leucine-binding pocket. Importantly, the study also identified the binding site for GATOR2 in close proximity to the leucine-binding pocket of Sestrin2 [84]. It is conceivable that similar to the nature of arginine bound-CASTOR1, leucine binding to Sestrin2 induces conformational changes of the structure adjacent to the leucine-binding pocket, which may cause the transformation of the moiety of the GATOR2 binding site, thereby disrupting the interaction between GATOR2 and Sestrin2. Together, these studies indicate that Sestrin1 and Sestrin2 are physiological cytosolic leucine sensors that inhibit mTORC1 through GATOR2.

### 3.6. SZT2-Containing KICSTOR Recruits GATOR1 to the Lysosome and Inhibits Amino Acid-Induced mTORC1 Activation

Previous studies have indicated that GATOR1 inhibits RagA/B through its GAP activity while GATOR2 functions as a suppressor of GATOR1 through unknown mechanisms [63] (Figure 1). It has not been clearly understood how RagA/B, which lacks membrane-anchoring motifs are regulated by GATOR1. Two recent independent studies have identified that seizure threshold 2 homolog (SZT2) or the KICSTOR complex consisting of kaptin (KPTN), integrin alpha phenylalanyl-glycyl-glycyl-alanyl-prolyl (FG-GAP) repeat containing 2 (ITFG2), chromosome 12 open reading frame 66 (C12 or f66) and SZT2, plays an important role in tethering GATOR1 to the lysosome, thereby inhibiting the activity of RagA/B and mTORC1 [85,86]. Wolfson et al. identified that the SZT-containing KICSTOR complex localizes on the lysosome and interacts with GATOR1 independently of amino acid availability [85] (Figure 1). Deletion of any of the KICSTOR components blocks lysosomal localization of GATOR1 and disperses it throughout the cytoplasm without affecting levels of GATOR2 lysosomal localization. GATOR1 fails to interact with its substrates the Rag GTPases as well as its regulator, GATOR2 in cells lacking an intact KICSTOR complex. Importantly, as expected, in KICSTOR-deficient cells, amino acid deprivation fails to block lysosomal mTORC1 localization and activity. Thus, the study indicated that KICSTOR is an important scaffolding protein complex that tethers GATOR1 to the lysosomal membrane thereby not only inhibiting RagA/B activity but also maintaining intact amino acid-sensing mechanisms through CASTOR– and Sestrin–GATOR2 pathways (Figure 1). 

In a parallel study from Li’s group, STZ2 was also identified as a key interacting protein with both GATOR1 and GATOR2 [86]. Consistent with the observations reported in the study by Wolfson et al., amino acid deprivation fails to diminish lysosomal mTORC1 localization and activity in cells lacking STZ2. However, Peng et al. demonstrated that ablation of STZ2 diminishes both GATOR1 and GATOR2 localization on the lysosome [86]. Furthermore, ablation of either GATOR1 or GATOR2 also reduces lysosomal STZ2 localization, indicating that the integrity of the STZ2-orchestrated GATOR1–GATOR2 (SOG) complex is necessary for lysosomal localization of both GATOR complexes as well as STZ2 and for intact amino acid sensing to mTORC1 signaling. Intriguingly, while ablation of WDR59, a component of GATOR2, strongly inhibits amino acid-induced mTORC1 activation, artificially tethering WDR59 (lyso-WDR59) to the lysosomal membrane inhibits amino acid-insensitive mTORC1 activation in GATOR1/STZ2 or GATOR2/STZ2 deficient cells. These observations suggest that lysosomal WDR59 exerts an unexpected inhibitory function in the regulation of mTORC1 activity. Given that Sestrin2 interacts with GATOR2 under amino acid starvation conditions, and the lysosome-targeted Sestrin2 (lyso-Sestrin2) sufficiently inhibits mTORC1 activity in cells lacking the SOG complex, the study proposed that WDR59-contaning GATOR2 complex may have a key scaffolding role in recruiting Sestrin2 to inhibit RagA/B–mTORC1 activity independently of GATOR1. Indeed, these results support the idea that Sestrin2 functions as the guanine nucleotide dissociation inhibitor (GDI) for RagA/B through a putative GDI motif of Sestrin2 as previously proposed by the same group [87]. However, two recent independent Sestrin2 structure studies demonstrated that two of three key charged residues important for Sestrin2’s GDI activity are buried inside their structure, and Sestrin2 shows no structural similarity to known GDI proteins [84,88]. Thus, it remains unclear as to whether lyso-Sestrin2 inhibits mTORC1 activity in SGO-deficient cells through its GDI activity toward RagA/B. Although these two studies proposed slightly different models in terms of the role of SZT2/KICSTOR in the regulation of lysosomal GATOR2 localization, both studies demonstrated that the SZT2 or SZT2-containing protein complex, KICSTOR, is an essential component, which works cooperatively with GATORs and functions upstream of Rag A/B in the amino acid sensing pathway for the regulation of mTORC1. In addition, these studies highlighted aberrant activation of mTORC1 as a potential pathomechanism underlying the onset and development of epilepsy as well as macrocephaly since mutations in SZT2 and other components of KICSTOR (KPTN) have been identified in patients with these disorders [89,90,91,92,93]. In support of this idea, epileptic seizure or macrocephaly are major symptoms seen in patients with tuberous sclerosis complex (TSC) mutations in either TSC1 or TSC2 [94], or Cowden syndrome with PTEN mutations [95], respectively. 

### 3.7. The FLCN–FNIP Complex Functions as a GAP for Rag C/D

The activation of mTORC1 on the lysosomal membrane is regulated through not only RagA/B but also RagC/D. The active Rag complex is established by the hetero-dimerization of GTP-bound RagA/B and GDP-bound RagC/D. Recent studies identified that folliculin (FLCN) and its binding partner, FLCN interacting protein 1 (FNIP1) and 2 function together as a specific GAP for RagC/D [96,97] (Figure 1). The FLCN–FNIP protein complex is evolutionarily conserved from yeast to mammal. Importantly, loss of function mutations in the *FLCN* gene cause Birt–Hogg–Dube (BHD) syndrome, which is characterized by the formation of benign or malignant tumors in hair follicles (fibrofolliculomas), kidney, and lung, suggesting that FLCN is a tumor suppressor [98,99]. Tsun et al. demonstrated that the FLCN–FNIP complex localizes on the lysosome in an amino acid sensitive manner: amino acid starvation stimulates its lysosomal localization whereas amino acid stimulation dissociates the FLCN–FNIP complex from the lysosome [96]. Accordingly, the FLCN–FNIP complex preferentially interacts with the Rag heterodimer under amino acid starvation conditions [100]. It remains elusive why the FLCN–FNIP complex, which activates the Rag heterodimer, resides on the lysosomal surface under amino acid starvation conditions. However, the fact that FLCN functions as a GAP for RagC/D indicates that the FLCN–FNIP complex is a key activator of the Rag heterodimer and mTORC1. Thus, it also remains unclear how the FLCN–FNIP complex functions as a tumor suppressor. Intriguingly, while in most cell-based systems, acute loss of FLCN inhibits mTORC1 activation [101,102,103], ablation of FLCN in tissues causes the enhancement of mTORC1 activity in vivo [104,105,106,107]. These seemingly inconsistent observations suggest that other compensatory mechanisms for RagC/D activation may exist [108] and/or FLCN may have other biological functions that suppress tumorigenesis. How the FLCN–FNIP senses amino acids or the existence of upstream regulators of the FLCN–FNIP complex in amino acid signaling remains unknown. Han et al. previously reported that leucyl-tRNA synthetase (LRS) functions as a specific GAP for RagD by sensing cellular leucine [108]. However, the possibility of LRS as a GAP for RagD has been questioned by the study reported by Tsun et al. [96]. Instead, a more recent study demonstrated that LRS stimulates vacuolar protein sorting 34 (VPS34), an evolutionarily conserved class III-PI3K, which is known to activate mTORC1, in response to leucine availability [109]. 

## 4. The Spatial Regulation of TSC through Akt and Amino Acids

**Figure 2:**
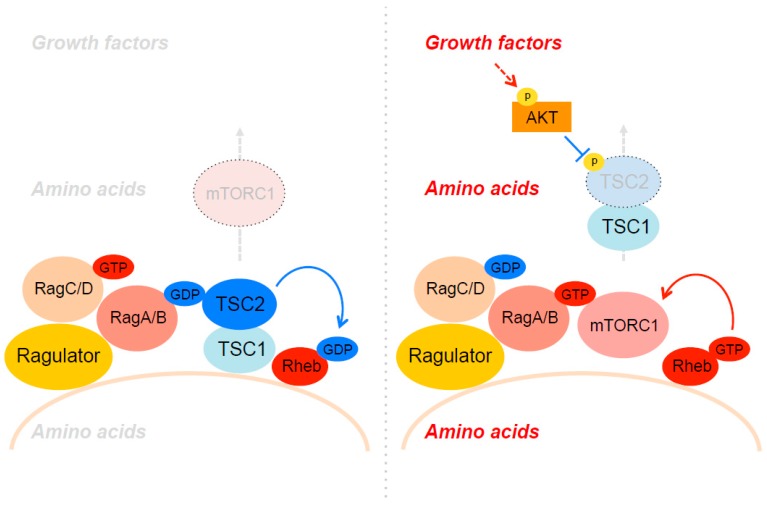
Spatial regulation of the tuberous sclerosis complex (TSC) complex on the lysosomes.The TSC complex preferentially interacts with both GDP-bound RagA and Rheb on the lysosomal membrane under growth factor and amino acid-deficient conditions. Upon growth factor and amino acid stimulation, TSC2 is phosphorylated by Akt and dissociates from the lysosomal membrane leading to the activation of Rheb.

mTORC1 is recruited to the lysosomal membrane through Rag GTPases in response to amino acid availability. Subsequently, lysosomal mTORC1 is directly activated by Rheb, which is inhibited by TSC2, a specific GAP for Rheb. While it has been well demonstrated that active Akt phosphorylates and inhibits TSC2 GAP activity thereby stimulating the Rheb–mTORC1 pathway [110,111], the molecular mechanisms by which Akt-induced TSC2 phosphorylation inhibits its GAP activity are still not well understood. Strikingly, a recent paper from Manning’s group revealed that the phosphorylation of TSC2 by Akt strongly induces the dissociation of the TSC complex from the lysosome [27]. In contrast, growth factor starvation or specific Akt inhibition strongly induces lysosomal localization of TSC2. Artificially tethering TSC2 to lysosomes (lyso-TSC2) confers mTORC1 activity insensitive to growth factor stimulation. Taken together, Akt stimulates Rheb–mTORC1 activity by repelling the TSC complex from lysosomal membranes through its phosphorylation of TSC2 (Figure 2). Interestingly, Rheb is required for lysosomal TSC localization as deletion of Rheb or disruption of lysosomal Rheb with a farnesyltransferase inhibitor disperses the TSC complex throughout the cytoplasm even under growth factor starvation conditions. Intriguingly, the TSC complex purified from serum-starved cells shows higher affinity to GDP-loaded Rheb than GTP-loaded Rheb, a property unusual among Ras family GAPs. It is possible that non-phosphorylated TSC2 or components in the TSC complex such as TSC1 and TBC1D7 may act as a GDI to block nucleotide exchange of GDP–Rheb on the lysosome under growth factor-deficient conditions. 

Teleman’s group also reported that the spatial regulation of the TSC complex is critical for the regulation of mTORC1 activity [43]. However, they demonstrated that lysosomal localization of the TSC complex is regulated by amino acids (Figure 2). Under amino acid-deprived conditions, the GDP-bound form of RagA strongly binds to TSC2 and recruits the TSC complex to lysosomes, thereby inhibiting Rheb–mTORC1 activity. Consistently, ablation of RagA/B or GATOR1, a RagA/B GAP, blocks lysosomal localization of the TSC complex even under amino acid-deprived conditions. Interestingly, in TSC2-deficient cells, mTORC1 remains localized on the lysosome in a manner dependent on active Rheb under amino acid-deprived conditions. Taken together, these observations suggest that mTORC1 localizes on the lysosomal membrane through both active GTP-bound Rag and Rheb under amino acid and growth factor enriched conditions, whereas the TSC complex takes over the place through inactive GDP-bound Rag and Rheb under amino acid and growth factor-deficient conditions (Figure 2). This swapping between mTORC1 and the TSC complex through “dual anchoring” mechanism explains how growth factor and amino acid stimulation impinge on lysosomal membranes and coordinately turn on or off the activity of mTORC1. In line with the above model, Carroll et al. reported that growth factors and arginine, a key amino acid that activates mTORC1, induce dissociation of the TSC complex from lysosomes [44]. Interestingly, arginine directly blocks the association between TSC2 and Rheb in vitro. These observations suggest that arginine contributes to mTORC1 activation through its direct action on the TSC complex–Rheb interaction in addition to the activation of Rag small GTPases via SLC38A9 and CASTORs. It is anticipated that more amino acid-sensing molecules and mechanisms likely exist and will be revealed by undergoing and future studies.

## 5. Concluding Remarks

The ability of cells to respond appropriately to nutrient availability is of fundamental importance for adaptation to the environment. In response to nutrient availability or metabolic stresses, cells modulate the rate of anabolism or catabolism, respectively. In these processes, mTORC1 is a central player that induces cell growth and proliferation by activating protein, pyrimidine, and lipid biosynthesis. In addition, mTORC1 also plays a key role in suppressing autophagy, a major cellular catabolic process. In this review, we summarized current knowledge and understanding of amino acid-sensing mechanisms that regulate mTORC1, especially on the lysosomal membrane of mammalian cells. Although emerging evidence indicates that leucine/arginine–Rag-dependent recruitment of mTORC1 to the lysosome and its subsequent binding to Rheb plays a pivotal role in the activation of mTORC1, it has not been clearly understood how other amino acids such as glutamine and lysine that have a potential to activate mTORC1 are sensed and lead to its activation. Glutamine has been reported to function as an efflux solute to increase influx of leucine through the SLC7A5–SLC3A2 heterodimeric antiporter expressed on the plasma and lysosomal membrane [112]. In addition, it has been reported that α-Ketoglutarate, a glutamine metabolite, stimulates the Rag–mTORC1 pathway [113]. However, glutamine is able to induce lysosomal mTORC1 localization and its activation in RagA/B knockout cells [62]. Thus, it appears that glutamine acts on multiple targets upstream of mTORC1 to enhance its activity. 

Furthermore, it remains unclear where and how mTORC1 or the TSC complex localizes in the cytoplasm under amino acid-insufficient or -enriched conditions, respectively. Recent studies indicated that mTORC1 is tethered to or incorporated into unknown cytoplasmic punctate structures or stress granules under amino acid-deficient or severe metabolic stress conditions, respectively [26,114,115]. These observations suggest that there are undefined amino acids and/or growth factor-sensing mechanisms, which may relieve mTORC1 from stress-related compartments and support its trafficking to the lysosomes for reactivation. 

In addition, it is also not well understood where mTORC1 phosphorylates its distinct substrates, which are expressed in different cellular compartments. Indeed, a recent study demonstrated that active mTORC1 phosphorylates its substrates in multiple cellular compartments. Using the subcellularly targeted specific mTORC1 reporter system, it was found that mTORC1 is able to phosphorylate its substrates not only in cytosol and on the lysosomal membrane but also in the nucleus and plasma membrane. Interestingly, while growth factors widely enhance mTORC1 activity throughout these subcellular compartments, leucine-induced mTORC1 activity is more restricted to the lysosomal membrane and nucleus [116]. These observations raise the possibility that mTORC1 may be delivered to these compartments after its activation on the lysosome [58] or activated in the nucleus by amino acids and growth factors through undefined machineries. Answering these questions would provide further insights into the molecular mechanisms underlying mTORC1 regulation, and help to facilitate the identification of potential targets for treating mTORC1-associated health problems.
